# G protein-coupled receptor autoantibody expression patterns in adults with decelerated biological aging mirror pediatric profiles

**DOI:** 10.1186/s12865-026-00862-4

**Published:** 2026-06-04

**Authors:** Krystallenia Paniskaki, Ulrik Stervbo, Sarah Goretzki, Doreen Hufnagel, Moritz Anft, Harald Heidecke, Timm H. Westhoff, Hana Rohn, Ursula Felderhoff-Mueser, Oliver Witzke, Christian Dohna-schwake, Nina Babel

**Affiliations:** 1 Center for Translational Medicine and Immune Diagnostics Laboratory, Medical Department I, Marien Hospital Herne, University Hospital of the Ruhr-University Bochum, Bochum, Germany; 2 Department of Infectious Diseases, West German Centre of Infectious Diseases, University Hospital Essen, University Duisburg-Essen, Essen, Germany; 3 Fifth Department of Medicine, University Medical Centre Mannheim, University of Heidelberg, Mannheim, Germany; 4 Department of Pediatrics I, University of Duisburg-Essen, Children´S Hospital Essen, Essen, Germany; 5 CellTrend GmbH, 14943 Luckenwalde, Germany; 6 Medical Department I, Marien Hospital Herne, University Hospital of the Ruhr-University Bochum, Herne, Germany; 7 Center for Translational Neuro- and Behavioral Sciences C-TNBS, University Duisburg-Essen, Essen, Germany; 8 BIH Center for Regenerative Therapies (BCRT) Berlin, Berlin Institute of Health at Charité – University Clinic Berlin, Berlin, Germany

**Keywords:** G protein-coupled receptors, GPCR, GCPR, Autoantibodies, Biological age, Aging

## Abstract

**Background:**

G protein-coupled receptors (GPCRs) represent the largest and most diverse family of membrane proteins. In addition to their endogenous ligands, functional natural autoantibodies (Aab) have also been shown to bind and modulate GPCRs. Aab concentration is known to increase with chronological age, and several studies demonstrated an association of certain GPCR-binding Aab with chronic inflammation, concretely with specific autoimmune diseases in adults. However, prevalence of GPCR-Aab in pediatric population is not known so far.

**Methods:**

In this descriptive study, we investigated the levels of Aab targeting 14 distinct GPCRs across individuals (pediatric, *n* = 83, adults *n* = 198) taking into consideration chronological and biological age, as defined by PhenoAge clock.

**Results:**

Our findings suggest, that anti-GPCR Aab are present from childhood through adulthood; however, their expression patterns undergo significant changes over the course of life. Specifically, we observed a significant increase in Aab targeting ACE2, CXCR3, and BDKRB1 in adults and older individuals. Conversely, concentrations of Aab against ATR1, ADRA1A, ADRB2, and ETAR were significantly higher in children compared to adults. We also assessed the impact of age acceleration on Aab levels, defined by a positive Δ age score based on the PhenoAge clock. Distinct GPCR Aab expression signatures correlated with accelerated aging, differing notably from patterns seen in pediatric and adult cohorts. Concretely, the expression profile of GPCR Aab in -Δage adults exhibits similarity to that observed in the pediatric cohort. Furthermore, we found a concordant presence of CXCR3-Aab and expansion of CXCR3 + T cells with increased chronological age.

**Conclusions:**

Because systemic chronic inflammation is a known driver of accelerated aging, we hypothesize that the altered Aab expression profiles observed in biologically older adults may reflect this underlying inflammatory state. Although future research will be essential to evaluate the therapeutic potential of targeting these GPCRs in the context of inflammaging and autoimmune pathophysiology, chronological as well as biological age should be considered when assessing GPCR-Aab patterns.

**Supplementary Information:**

The online version contains supplementary material available at 10.1186/s12865-026-00862-4.

## Background

Natural autoantibodies (Aab) function as part of the innate immune system, have homeostatic functions such as clearing apoptotic cells and oxidized proteins [1–3]. Sometimes termed physiological or naturally occurring Aab, they are the most abundant of all Aab and are typically polyreactive and bind with low affinity [4–6]. This contrasts with pathogenic Aab, which are derived through affinity maturation and have high affinity and specificity [7]. Natural Aab contribute to the establishment and maintenance of immune memory in a manner, that is distinct from classical immune reactions and may be used as predictors of future disease in presently healthy individuals [8, 9]. Natural Aab are acquired in early childhood [10] and are also found in healthy pediatric individuals [8, 11–14].

G protein-coupled receptors (GPCRs) comprise the largest and most diverse family of integral membrane proteins that participate in different physiological processes such as the regulation of the nervous and immune system [15, 16]. Besides the endogenous ligands of GPCRs, functional Aab are also able to bind GPCRs, triggering or blocking intracellular signalling pathways, resulting in agonistic or antagonistic effects respectively [17, 18]. However, serum levels of GPCR Aab of healthy individuals is not per definition lower than in that of patients with autoimmune disorders [4, 19]. Besides the physiological roles, the levels of Aab against GPCRs are altered in pathological conditions, and both increased and decreased concentrations correlate with the development or progress of immune-mediated disorders as well as disease specificity [20].

High levels of Aab against the muscarinic acetylcholine receptor M3 as well as those targeting endothelin receptor type A (ETAR) and type 1 angiotensin II receptor (AT1R) have implications in the pathogenesis of rheumatic diseases such as Sjörgen syndrome [21] and systemic sclerosis [20, 22]; AT1R and ETAR determine the extent of vasculopathy in systemic sclerosis [22] and are also associated cardiovascular disease [23–25]. Dysregulation of Renin–Angiotensin–Aldosterone System Aab was suggested as a new mechanism, that can contribute to Parkinson disease progression, as higher serum AT1 and ACE2 Aab were found among patients with Parkinson disease [26].

As mentioned above, elevated titers of Aab against GPCRs have been demonstrated and correlated with the pathogenesis of age-related diseases. It is therefore reasonable to hypothesize, that the concentration of GPCR-targeting Aab increases with age, potentially reflecting the phenomenon of "inflammaging," an acknowledged hallmark of the aging process [27]. Previous studies have investigated age-related changes in natural Aab levels [4, 8, 11, 12, 19, 28, 29]; however, those studies did not specifically focus on Aab directed against GPCRs, nor did they include pediatric populations. Therefore, in this descriptive study, we examine the levels of Aab targeting 14 distinct GPCRs, two intracellular proteins, and two transmembrane receptors across two distinct age groups: pediatric and adult.

## Materials and methods

### Aab against CGP-receptors & absolute IgG concentration

Human IgG Aab against 14 different GPCRs (ATR1, ATR2, MAS1, BDKRB1, ADRA1A, ADRB2, CXCR3, ETAR, ETBR, M5R, PAR1, PAR2), two proteins (ACE2, ANXA2) and the transmembrane receptors ANXA2R and STAB1 (Autoantibodies dataset and full name in Table S1) were detected from frozen serum using commercial ELISA kits (CellTrend, Luckenwalde, Germany) according to the manufacturer’s instructions as previously described [30].

Absolute IgG concentration was measured via immunoturbidimetry from Central Laboratory of University Duisburg-Essen, Essen, Germany. A sample containing human IgG was suitably diluted and reacted with a specific antibody to form a precipitate, which was measured at 340 nm. The IgG concentration was determined using a calibration curve.

### SARS-CoV-2 nucleocapsid IgG

Human IgG antibodies against the SARS-CoV-2 nucleocapsid protein were detected using commercial ELISA kits (EUROIMMUN) according to the manufacturer’s instructions.

### PBMCs isolation

As previously described [31], peripheral blood was collected in S-Monovette K3 EDTA tubes (Sarstedt). The samples were diluted 1:1 with PBS/BSA (Gibco) and carefully layered over 15 mL of Ficoll-Paque Plus (GE Healthcare). Following centrifugation at 800 × g for 20 min at room temperature, PBMCs were isolated, washed twice with PBS/BSA, and stored at − 80 °C until further use. For downstream applications, cryopreserved PBMCs were thawed by incubating cryovials in a 37 °C bead bath for 2–3 min. Cells were then washed twice with prewarmed RPMI 1640 medium (Life Technologies) containing 1% penicillin–streptomycin–glutamine (Sigma-Aldrich) and 10% fetal calf serum (PAN-Biotech), and subsequently incubated overnight at 37 °C.

### Flow cytometry- characterization T cells

Thawed and rested overnight PBMCs were plated in 96-U-Well plates in RPMI 1640 media (Life Technologies). The PBMCs were stained with optimal concentrations of antibodies for 10 min at room temperature in the dark. Stained cells were washed with PBS/BSA and were immediately acquired on a CytoFLEX flow cytometer (Beckman Coulter) (gating strategy Fig. 2A). Fluorescence minus one controls were used for optimal gating of chemokine receptor CXCR3 (CXCR3) populations. No modification to the compensation matrices was required throughout the study. Detailed listing of the antibody panel (extracellular staining) for the characterization of T cells is presented in supplementary table S2.

### Statistics

Statistical analysis was performed using GraphPad Prism (v7) and R (v 4.2.2). Categorical variables are summarized as numbers and frequencies; quantitative variables are reported as median and interquartile range. Normality tests were performed with the Shapiro–Wilk Test. All applied statistical tests are two-sided. Kruskal–Wallis Test and Mann–Whitney-Test were applied to perform comparisons. Correlational analysis was performed with Pearson test. Biological sex was compared using two-tailed Fisher’s exact test. p values below 0.05 were considered significant; only significant p values are reported in the figures. p values were not corrected for multiple testing, as this study was of an exploratory nature.

### Calculation of PhenoAge score

Aging clocks are machine learning models designed to identify patterns in molecular features across extensive sample cohorts, which can subsequently be used to estimate the biological age of the sample origin [32]. It has been widely hypothesized that this estimated age can serve as a measure of an individual’s biological age, and that the difference between estimated and actual chronological age, called ‘Δ age’, reflects variation in their past rate of aging [32–34]. These hypotheses have been supported by observations, that positive Δ age, termed age acceleration, is associated with systemic inflammation termed as ``inflammaging``, increased cardiovascular disease risk as well as other age-related diseases [32, 35].

Levine et al. [36] built the PhenoAge clock, and while this biomarker was developed using data from whole blood, it correlates strongly with age in every tissue and cell. This biomarker of aging, among others [37], is able to capture risks for diverse outcomes across multiple tissues and cells and provide insight into important pathways in aging. Furthermore, is associated with increased activation of pro-inflammatory and interferon pathways, and decreased activation of transcriptional/translational machinery, DNA damage response, and mitochondrial signatures. It is based on a linear combination of chronological age and nine clinical parameters associated with mortality risk [32, 36].

To assess biological aging, we calculated PhenoAge using the mathematical equation previously described [38] (fig. S1). The calculation incorporates several clinical biomarkers, including Albumin (g/L), Creatinine (µmol/L), Glucose (mmol/L), C-reactive protein (mg/dL), Lymphocyte percentage, Mean cell volume (fL), Red cell distribution width percentage, Alkaline phosphatase (U/L), and White blood cell count (10^3 cells/µL), along with chronological age. Each parameter was measured using standard clinical laboratory techniques. The resulting PhenoAge score is expressed in years. Participants were then stratified based on Δage into two groups: positive Δage (+ Δage), including those with Δage > 0, and negative Δage (− Δage), including those with Δage < 0. Positive Δage is termed as age acceleration, while negative Δage is termed as age deceleration.

## Results

### Characterization of the study group

We included serum samples from healthy adult individuals recruited prior to COVID-19 pandemic (between 2017 and 2019) and during the pandemic (between September 2020 and December 2021). Samples were collected from healthy pediatric and adult participants who were SARS-CoV-2-unexposed (nucleocapsid IgG negative) and PCR-negative at the time of recruitment for control purposes. Convalescent individuals with asymptomatic or mild COVID-19 infection were also included; these samples were obtained from the outpatient clinics of the Department of Pediatrics I and the Department of Infectious Diseases at University Hospital Essen. Individuals with long COVID syndrome were excluded from participation. A minimum convalescence period of 12 weeks was required for the convalescent individual group at the time of recruitment. Demographic and clinical characteristics of the pediatric and adult patients included in the study are provided in Table 1. The study group comprised of 198 adults (≥ 18 years old) and 83 pediatric subjects (< 18 years old). The adult cohort comprised of 49% (*n* = 97) female and 51% (*n* = 101) male participants, whereas the pediatric cohort of 45% (*n* = 37) female and 55% (*n* = 46) male participants and showed no statistical sex difference (Fisher´s exact test, *p* = 0.05155).

**Table 1:** Demographic and clinical characteristics of the study cohorts

	Children (*N* = 83)	Adults (*N* = 198)	*p* value
Age years -median (range)	11 (1–17)	59 (18–92)	< 0.0001
Female gender N (%)	37 (45)	97 (49)	0.05155
SARS-CoV-2 unexposed*^+^	43	73	N/A
COVID-19 Severity N (%) ^+#^			
*Asymptomatic N*	40	57	N/A
*Mild N*	0	68	N/A

^+^SARS-CoV-2 PCR negative at recruitment

^#^A minimum convalescence period of 12 weeks was required at the time of recruitment

^*^Nucleocapsid IgG negativ

### GPCR Aab prevalence changes with age

**Fig. 1:**
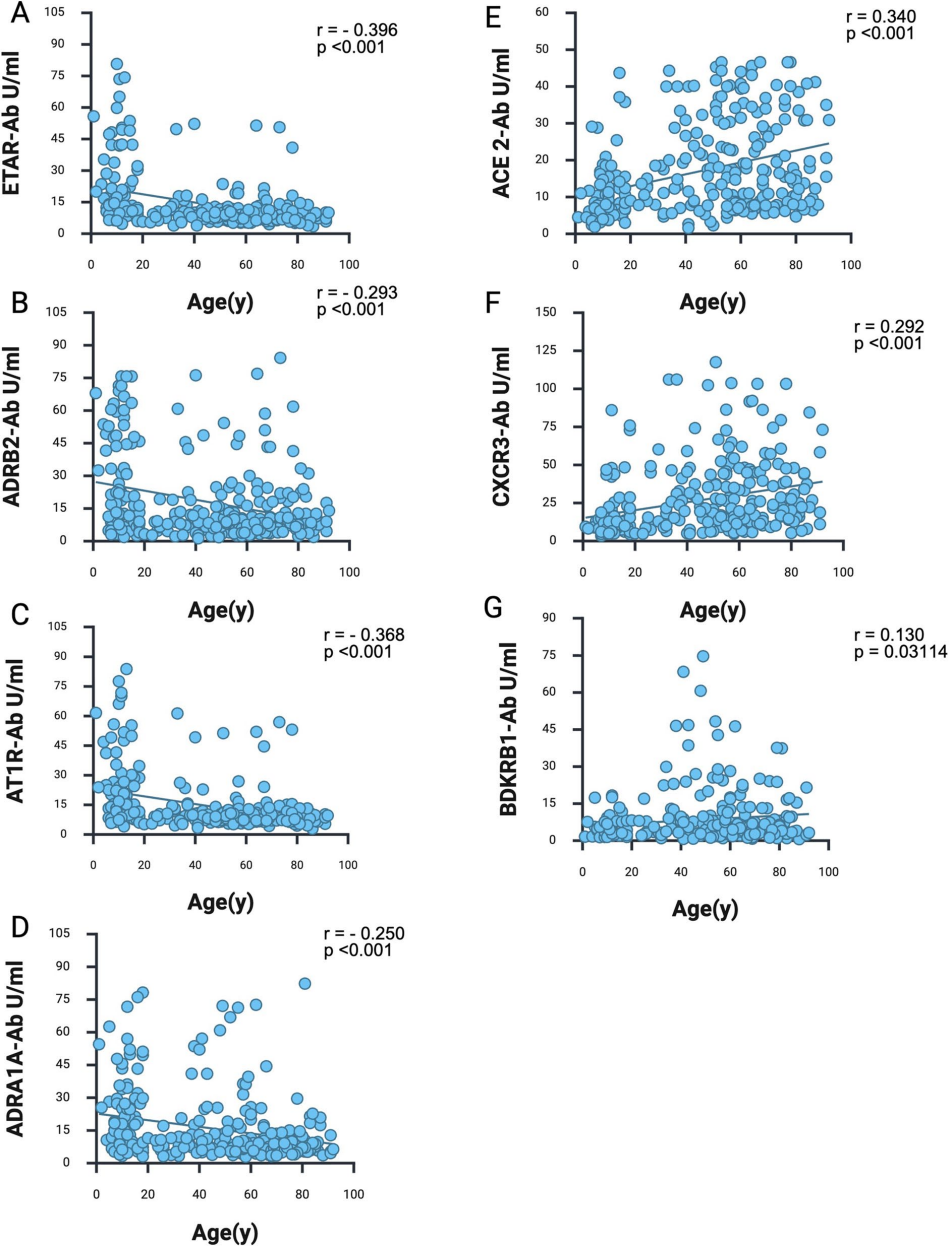
GPCR Aab prevalence changes with age. **A**-**D** Age-adjusted analysis of ATR1-, ADRA1A-, ADRB2-, and ETAR-Aab titers. **E**–**G** Age-adjusted analysis of ACE2, CXCR3 and BDKRB1-Aab titers. the data were log transformed, assigning a value of zero for those with a value below the detection limit. The data were analyzed using linear regression. *p* < 0.05 was considered significant

In order to exclude influence of age on the absolute IgG concentration in our cohort, we measured the absolute IgG titers on 31 pediatric and 17 adult subjects. The absolute IgG titers did not differentiate between adults and pediatric subjects (Mann–Whitney-Test, *p* > 0.05) (fig. S2). Correlational analysis between age and concentration of GPCR Aab revealed statistically significant differences influenced by age. ATR1-, ADRA1A-, ADRB2-, ETAR Aab concentration was statistically significantly higher among children compared to adults (Fig. 1A-D & S3), while we found a weak negative correlation between age and the concentration of ATR1-, ADRA1A-, ADRB2-, ETAR Aab (Fig. 1A-D). On the contrary ACE2, CXCR3 and BDKRB1-Aab titers are statistical significantly increasing with age (Fig. 1E–G & S3). Antibody titers against ATR2, MAS1, ADRB2, ETBR, M5R, PAR1, PAR2 and ANXA2R showed no statistical difference among the pediatric and adult cohort (fig. S3). Correlational analysis of biological sex with Aab concentration showed no influence of sex in concentration of GPCR Aab (fig. S4).

Taken together, these results suggest that natural Aab are present from childhood to adult life, however the expression patterns are significantly modified over the course of life with ATR1, ADRA1A, BDKRB1, ETAR Aab being predominant in early life and decreasing over the course of life. Furthermore, we observe a significant increase of ACE2, CXCR3 and BDKRB1 Aab in adulthood and later life. Sex does not seem to affect the concentration of GPCR Aab in our cohort.

### Concordant prevalence of CXCR3-Aab and expansion of CXCR3 + T cells with increased chronological age

**Fig. 2:**
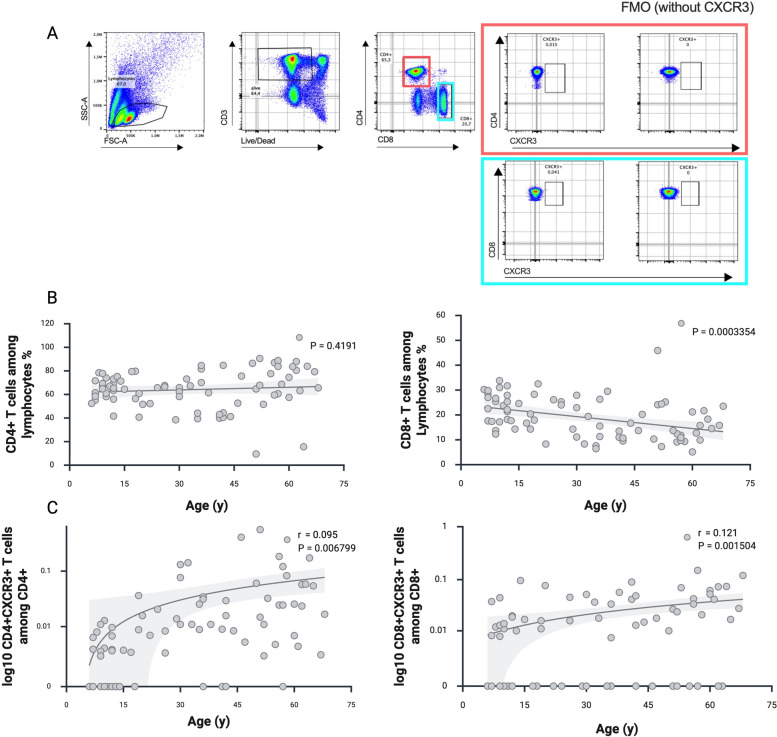
Expansion of CXCR3 + T cells with increased chronological age. **A** Flow cytometry gating strategy for identification of activated/migratory T cells. Thawed and rested overnight PBMCs were stained with optimal concentrations of antibodies. Living single lymphocytes were analyzed for expression of CD3, CD4, and CD8. The expression of the activation and migration marker CXCR3 was assessed on CD4 + (orange boxes) and CD8 + (blue boxes) single positive T cells. Fluorescence minus one control (FMO) were used for optimal gating of CXCR3 + population. **B** Analysis of absolute CD4 + and CD8 + T cells regarding age. **C** Analysis of CD4 + CXCR3 + and CD8 + CXCR3 + T cells regarding age. For the analysis of CD4 + CXCR3 + and CD8 + CXCR3 + T cells, the data were log transformed, assigning a value of zero for those with a value below the detection limit. The data were analyzed using linear regression. *p* < 0.05 was considered significant

Among other Aab, we found significantly increasing titers of CXCR3-Aab with age. CXCR3 receptor is activated by three interferon-inducible ligands CXCL9, CXCL10 and CXCL11 and plays an important role in T cell trafficking and function [39–41]. Accumulating data indicate the crucial role of CXCR3 in directing the migration of inflammatory CD4 + but mainly CD8 + T-cells [41–43]. As functional analysis of the GPCR Aab is challenging to pursue, we assessed the CXCR3 expression from CD4 + and CD8 + T cells via multiparameter flow cytometry. We found statistically significant increasing frequencies of CXCR3 + CD4 + and CXCR3 + CD8 + T cells with increasing age (Fig. 2B-C). The population of CXCR3 + CD4 + and CD8 + is rather small, however is significantly increasing with age. Correlational analysis between CXCR3 + T cells and CXCR3-Aab did not show any correlation pattern.

As CXCR3 and its ligands are considered part of an inflammatory chemokine system [44], the increase in CXCR3-Aab, along with higher frequencies of CXCR3⁺ T cells observed in our cohorts with chronological aging, may reflect a state of chronic inflammation.

### GPCR Aab expression-patterns in adults with decelerated biological aging mirror pediatric expression patterns

**Fig. 3:**
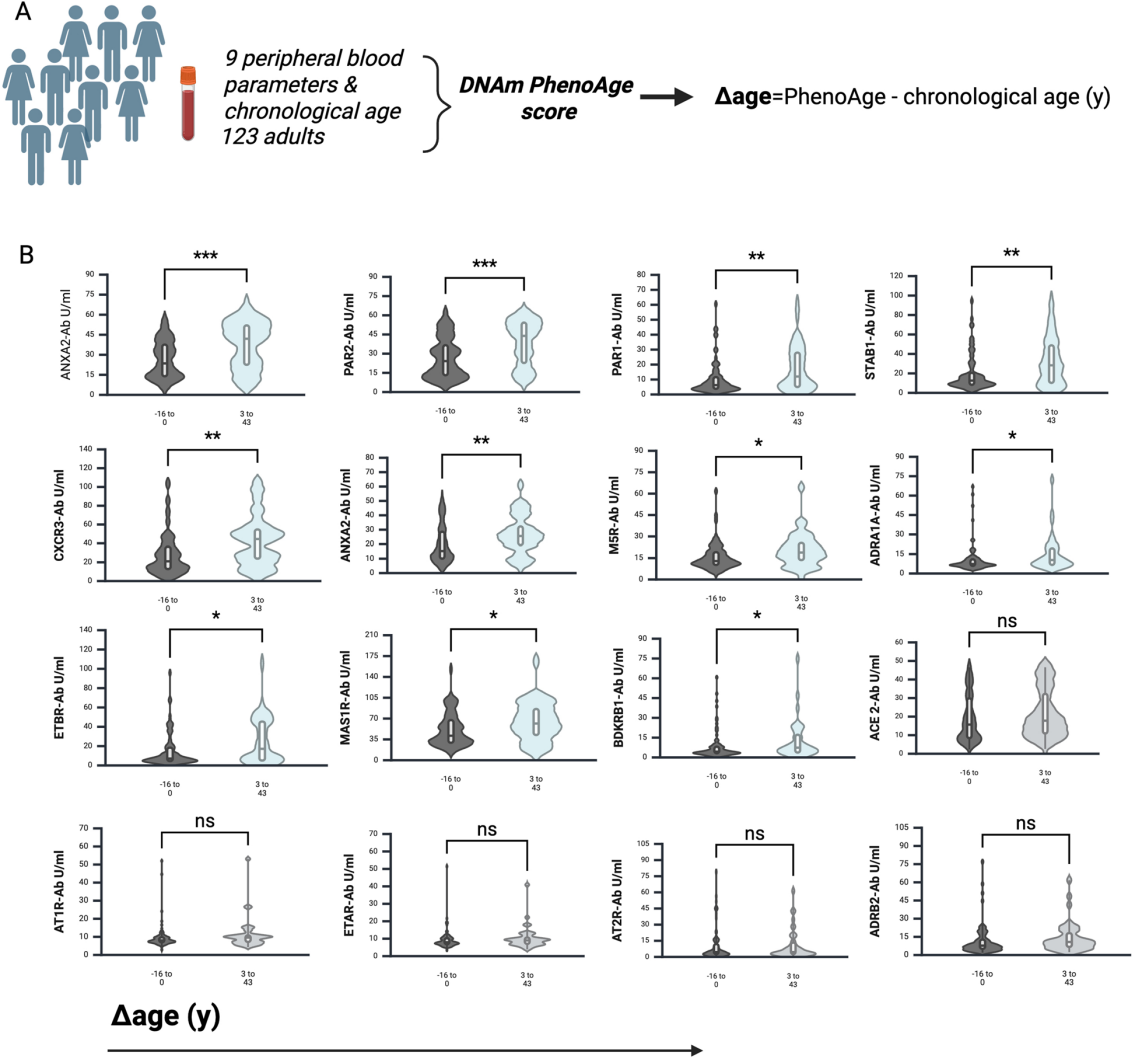
Signature patterns of GPCRs-Aab driven from accelerated aging. **A** The PhenoAge score was calculated for 123 adult participants. The difference between estimated and actual chronological age, called ‘Δ age’, reflects variation in their past rate of aging. **B** Analysis of anti-GPCR Aab concentrations of both study groups. Scatterplots show line at median. Unpaired data were compared with Mann–Whitney-test. *p* < 0.05 was considered significant, only significant p values are documented in the figures

We calculated the PhenoAge score of the 123 adult participants, with Δage as the difference to chronological age. Positive Δage is termed as age acceleration, while negative Δage is termed as age deceleration. The participants were subsequently separated by positive or negative Δage. The concentration of ANXA2, PAR2, PAR1, STAB1, CXCR3, ANXA2R, M5R, ADRA1A, ETBR, MAS1R and BDKRB Aab was significantly higher among the + Δage group. ATIR, ETAR, AT2R and ADRB2 Aab concentration was comparable in both groups (Fig. 3). These results demonstrate signature patterns of GPCRs Aab correlating with accelerated aging, which are distinguished from the expression patterns observed among the pediatric and adult cohort (fig S3).

**Fig. 4:**
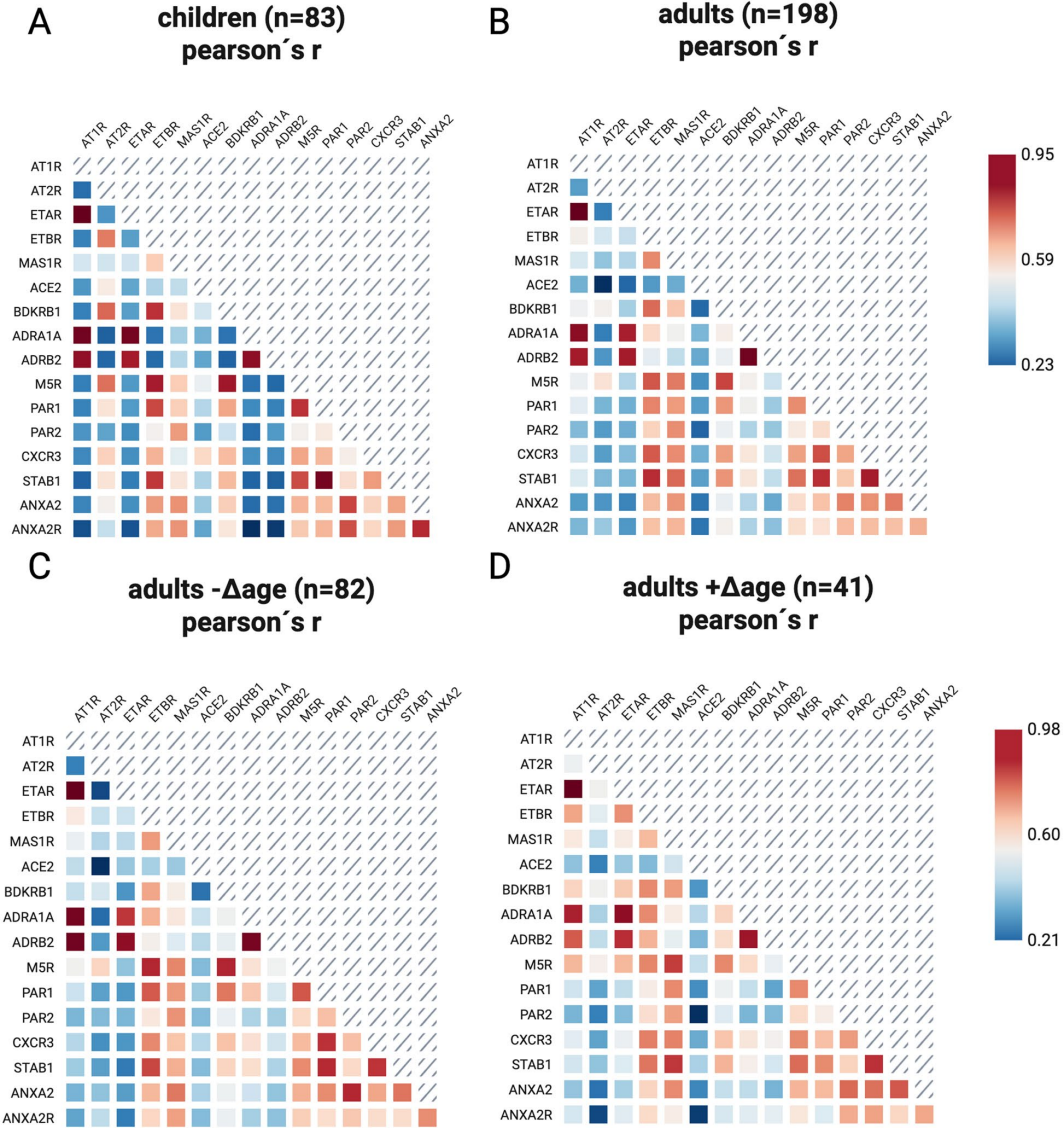
GPCR-Aab expression in -Δage adults mirrors pediatric patterns. Heatmap analysis of correlations of diverse anti-GPCR Aab among (**A**) children, (**B**) adults, (**C**) adults with negative Δ age, (**D**) adults with positive Δ age. Pearson’s r values are represented by varying shades of blue and red

We further explored possible correlations among the different GPCR Aab, taking into consideration the chronological age (adults and children) as a third parameter. We conducted bivariate correlation analyses of antibody concentrations (2 antibodies at a time) across four subcohorts: adults, children, adults with + Δage, and adults with − Δage. Figure S5 presents six representative plots illustrating these correlations. The correlation coefficients (r values) for each cohort are subsequently summarized in individual heatmap diagrams. Moderate to strong positive correlations of diverse GPCR Aab were observed between both cohorts with this trend being more prevalent in the adult’s cohort (Fig. 4A-B). Concretely ETBR, MAS1R, M5R, PAR1, CXCR3, Stabilin1 Aab correlated strongly positively with diverse GPCR Aab (fig. S6). We did not observe similar signatures among the pediatric cohort (fig. S6).

We further explored patterns of GPCR Aab correlations among the adult cohorts dividing the cohort once again based on positive or negative Δage. It is of interest, that almost the same pattern of strong positive correlations driven from ETBR, MAS1R, M5R, CXCR3, stabilin1 and ANXA2R Aab reappears among the + Δage but not among the -Δag cohort (Fig. 4C-D). Distinct GPCR Aab expression-patterns are present among adults with accelerated biological age. The expression profile of GPCR Aab in -Δage adults exhibits similarity to that observed in the pediatric cohort (Fig. 4 & fig. S6-7).

## Discussion

In this descriptive study, we explored the influence of biological and chronological age on concentration of GPCR Aab in a large cohort of pediatric and adult individuals. We found, that natural Aab are present in childhood and adult life, however the expression patterns are significantly altered over the course of life. We observed a significant increase of ACE2, CXCR3 and BDKRB1 Aab levels in adulthood and later life. However, ATR1-, ADRA1A-, BDKRB1-, ETAR-Aab levels are predominant in early life and decrease over the course of life. This observation stands in contrast to the prevailing notion, that concentration of natural Aab increases progressively with age [6, 45].

Age has a significant influence on absolute IgG concentrations. In pediatric populations, IgG levels increase with age, concretely, IgG concentrations in children are lower in infancy and gradually rise, often not reaching adult levels until adolescence or later [46, 47]. In adults, age-related changes persist, with some studies showing variations in IgG and its subclasses across age groups [48]. Furthermore, IgG accumulates in various tissues of mice and humans during aging [49]. It is therefore noteworthy, that in our study children exhibit higher concentrations of specific anti-GPCR IgG Aab compared to adults, although we found no differences in absolute IgG concentration between adult and pediatric cohorts. Higher anti-GPCR IgG Aab concentrations in children may reflect the dynamic maturation of the immune system during development. This period is characterized by increased B-cell and plasma cell activity, as well as heightened immune responsiveness to environmental antigens, infections, and vaccinations [50, 51].

Taken the above into consideration, we postulate, that alterations in GPCR expression, whether in surface density, activation threshold, or inhibition dynamics [4, 6], may vary across the human lifespan, contributing to the observed differences in anti-GPCR Aab profiles and immune function across lifespan. Similarly, the properties of their corresponding Aab, including binding affinity or structural conformation, may also undergo age-related changes [52].

No significant association was observed between Aab levels and biological sex. Consistent with our findings, Shome et al. reported no significant sex-related bias in serum Aab expression patterns [45]. However, other studies have described a slight influence of biological sex on the expression of natural Aab [4, 6, 53].

In addition to chronological age, we investigated the influence of biological age and age acceleration on Aab levels. To estimate biological age within the adult cohort, we employed the PhenoAge metric. Our findings reveal distinct GPCR Aab signatures associated with accelerated aging, which are clearly distinguishable from the developmental expression patterns described above. Overall, we identify two distinct Aab expression trajectories: one associated with physiological development from childhood to adulthood, and another linked to accelerated aging in adults with a positive Δ age score.

When accounting for chronological age, moderate to strong positive correlations among various GPCR Aab were observed across both cohorts, with this trend being more pronounced in the adult cohort. Of note is, that we observed near perfectly linear correlations between several Aab, most notably between ETAR and AT1R Aab. This strong association may be attributable to potential cross-reactivity. However, current molecular and immunological evidence does not support broad cross-reactivity based solely on short linear motifs. Literature indicates, that Aab against AT1R and ETAR are typically directed against conformational epitopes, often within the second extracellular loop of these GPCRs, rather than short linear motifs alone [54, 55]. Therefore, cross-reactivity between AT1R and ETAR Aab based solely on short linear motifs is unlikely, as antibody specificity is determined by conformational epitopes within the extracellular domains of these receptors [56, 57].

A similar pattern of strong positive correlations re-emerged specifically among adults with accelerated biological aging (+ Δage), but was absent in the -Δage group. Importantly, the GPCR Aab expression patterns in -Δage adults more closely resembled those observed in the pediatric cohort. Cabral and colleagues identified correlational relationships among anti-GPCR-Aab targeting structurally and functionally related molecules, such as vascular, neuronal or chemokine receptors, describing these networks, as a natural component of the immune system homeostasis [4]. This balance may become dysregulated, due to inflammation, cell damage or other stimuli [19, 58]. Age acceleration is associated with systemic inflammation termed as ``inflammaging``, which is a recognized hallmark of the aging process [27], increased cardiovascular disease risk as well as other age-related diseases [32, 35–37]. According to our findings, we hypothesize, that this pronounced Aab correlational expression pattern observed in adults with accelerated biological aging may reflect this state of chronic inflammation.

Among other Aab, we found significantly increasing titers of CXCR3-Aab with age. As CXCR3 and its ligands are considered part of an inflammatory chemokine system [44], the increase in CXCR3-Aab, along with higher frequencies of CXCR3⁺ T cells observed in our adult cohort, may reflect an inflammatory state. Upregulation of the CXCR3 receptor has been associated with neuronal axonal damage secondary to inflammatory response of T cells a) during or after viral infection [43, 59] as well as b) in the frame of autoimmunity [60] mainly in multiple sclerosis patients [39, 61]. Accumulating data indicate the crucial role of CXCR3 receptor in directing the migration of inflammatory CD4 + but mainly CD8 + T-cells in an CXCR3-dependent manner [41–43].

Our study has limitations. Thus, we were unable to perform a functional analysis of GPCRs and their corresponding Aab. Future longitudinal investigations employing concurrent functional analyses of GPCRs and their corresponding Aab across pediatric and geriatric cohorts are warranted to elucidate the mechanistic basis of this functional switch. Taking into consideration the crucial role natural Aab play in maintaining the body homeostasis, the variation in their concentration at different age intervals may partially account for the immunological and pathological changes that occur in the elderly [1, 18]. Immune architecture is shaped by a range of factors, including vaccination history, chronic infections, and systemic inflammatory status. Accordingly, elucidating the influence of these variables, and their potential reciprocal interactions on GPCR AAb concentrations, remains of interest. Future studies will be critical for assessing the prognostic and therapeutic potential of these receptors as pharmacological targets in the contexts of senescence, inflammaging, and pathophysiology of autoimmunity.

## Conclusions

Our findings indicate that anti-GPCR Aab are present from childhood through adulthood; however, their expression patterns undergo significant changes over the course of life. We also assessed the impact of age acceleration, defined by a positive Δ age score based on the PhenoAge clock, on Aab levels. Distinct GPCR Aab expression signatures correlated with accelerated aging, differing from patterns seen in the pediatric and adult cohorts. Furthermore, we found a concordant prevalence of CXCR3-Aab and expansion of CXCR3 + T cells with increased chronological age. Because systemic chronic inflammation is a known driver of accelerated aging, we hypothesize that the altered Aab expression profiles observed in biologically older adults may reflect this underlying inflammatory state.

## Supplementary Information

Supplementary Material 1.

## Data Availability

The datasets used and/or analysed during the current study are available from the corresponding author on reasonable request.

## References

[1] Jazayeri MH, Pourfathollah AA, Rasaee MJ, Farhadi M, Zarei N, Jafari ME. The reactivity of human serum natural autoantibodies with certain autoantigens increases along with aging. Biomed Aging Pathol. 2013;3:115–8. 10.1016/j.biomag.2013.06.001.

[2] Elkon KB, Silverman GJ. Naturally occurring autoantibodies to apoptotic cells. Adv Exp Med Biol. 2012;750:14–26. 10.1007/978-1-4614-3461-0_2. 22903663 10.1007/978-1-4614-3461-0_2

[3] Chou MY, Fogelstrand L, Hartvigsen K, Hansen LF, Woelkers D, Shaw PX, et al. Oxidation-specific epitopes are dominant targets of innate natural antibodies in mice and humans. J Clin Invest. 2009;119:1335–49. 10.1172/JCI36800. 19363291 10.1172/JCI36800 PMC2673862

[4] Cabral-Marques O, Marques A, Giil LM, De Vito R, Rademacher J, Günther J, et al. GPCR-specific autoantibody signatures are associated with physiological and pathological immune homeostasis. Nat Commun. 2018;9:5224. 10.1038/s41467-018-07598-9. 30523250 10.1038/s41467-018-07598-9 PMC6283882

[5] Lund A, Giil LM, Slettom G, Nygaard O, Heidecke H, Nordrehaug JE. Antibodies to receptors are associated with biomarkers of inflammation and myocardial damage in heart failure. Int J Cardiol. 2017;250:253–9. 10.1016/j.ijcard.2017.10.013. 29046223 10.1016/j.ijcard.2017.10.013

[6] Nagele EP, Han M, Acharya NK, DeMarshall C, Kosciuk MC, Nagele RG. Natural IgG autoantibodies are abundant and ubiquitous in human sera, and their number is influenced by age, gender, and disease. PLoS One. 2013;8:e60726. 10.1371/journal.pone.0060726. 23589757 10.1371/journal.pone.0060726 PMC3617628

[7] Lutz HU, Binder CJ, Kaveri S. Naturally occurring auto-antibodies in homeostasis and disease. Trends Immunol. 2008;30:43–51. 10.1016/j.it.2008.10.002. 19058756 10.1016/j.it.2008.10.002

[8] Mirilas P, Fesel C, Guilbert B, Beratis NG, Avrameas S. Natural antibodies in childhood: development, individual stability, and injury effect indicate a contribution to immune memory. J Clin Immunol. 1999;19:109–15. 10.1023/a:1020554500266. 10226885 10.1023/a:1020554500266

[9] Scofield RH. Autoantibodies as predictors of disease. Lancet. 2004;363:1544–6. 10.1016/S0140-6736(04)16154-0. 15135604 10.1016/S0140-6736(04)16154-0

[10] Mouthon L, Lacroix-Desmazes S, Nobrega A, Barreau C, Coutinho A, Kazatchkine MD. The self-reactive antibody repertoire of normal human serum IgM is acquired in early childhood and remains conserved throughout life. Scand J Immunol. 1996;44:243–51. 10.1046/j.1365-3083.1996.d01-306.x. 8795718 10.1046/j.1365-3083.1996.d01-306.x

[11] Maddur MS, Lacroix-Desmazes S, Dimitrov JD, Kazatchkine MD, Bayry J, Kaveri SV. Natural antibodies: from first-line defense against pathogens to perpetual immune homeostasis. Clin Rev Allergy Immunol. 2020;58:213–28. 10.1007/s12016-019-08746-9. 31161341 10.1007/s12016-019-08746-9

[12] Lacroix-Desmazes S, Mouthon L, Coutinho A, Kazatchkine MD. Analysis of the natural human IgG antibody repertoire: life-long stability of reactivities towards self antigens contrasts with age-dependent diversification of reactivities against bacterial antigens. Eur J Immunol. 1995;25:2598–604. 10.1002/eji.1830250929. 7589132 10.1002/eji.1830250929

[13] Madi A, Hecht I, Bransburg-Zabary S, Merbl Y, Pick A, Zucker-Toledano M, et al. Organization of the autoantibody repertoire in healthy newborns and adults revealed by system level informatics of antigen microarray data. Proc Natl Acad Sci U S A. 2009;106:14484–9. 10.1073/pnas.0901528106. 19667184 10.1073/pnas.0901528106 PMC2732819

[14] IJspeert H, van Schouwenburg PA, van Zessen D, Pico-Knijnenburg I, Driessen GJ, Stubbs AP, et al. Evaluation of the antigen-experienced B-cell receptor repertoire in healthy children and adults. Front Immunol. 2016;7:410. 10.3389/fimmu.2016.00410. 27799928 10.3389/fimmu.2016.00410 PMC5066086

[15] Sutkeviciute I, Vilardaga JP. Structural insights into emergent signaling modes of G protein-coupled receptors. J Biol Chem. 2020;295:11626–42. 10.1074/jbc.REV120.009348. 32571882 10.1074/jbc.REV120.009348 PMC7450137

[16] Yang LK, Wang W, Guo DY, Dong B. Non-canonical signaling initiated by hormone-responsive G protein-coupled receptors from subcellular compartments. Pharmacol Ther. 2025;266:108788. 10.1016/j.pharmthera.2024.108788. 39722422 10.1016/j.pharmthera.2024.108788

[17] Yu X, Wax J, Riemekasten G, Petersen F. Functional autoantibodies: Definition, mechanisms, origin and contributions to autoimmune and non-autoimmune disorders. Autoimmun Rev. 2023;22:103386. 10.1016/j.autrev.2023.103386. 37352904 10.1016/j.autrev.2023.103386

[18] Skiba MA, Kruse AC. Autoantibodies as endogenous modulators of GPCR signaling. Trends Pharmacol Sci. 2021;42:135–50. 10.1016/j.tips.2020.11.013. 33358695 10.1016/j.tips.2020.11.013 PMC7880908

[19] Cabral-Marques O, Riemekasten G. Functional autoantibodies targeting G protein-coupled receptors in rheumatic diseases. Nat Rev Rheumatol. 2017;13:648–56. 10.1038/nrrheum.2017.134. 28855694 10.1038/nrrheum.2017.134

[20] Akbarzadeh R, Müller A, Humrich JY, Riemekasten G. When natural antibodies become pathogenic: Autoantibodies targeted against G protein-coupled receptors in the pathogenesis of systemic sclerosis. Front Immunol. 2023;14:1213804. 10.3389/fimmu.2023.1213804. 37359516 10.3389/fimmu.2023.1213804 PMC10285309

[21] Abe S, Tsuboi H, Kudo H, Asashima H, Ono Y, Honda F, et al. M3 muscarinic acetylcholine receptor-reactive Th17 cells in primary Sjögren’s syndrome. JCI Insight. 2020;5:e135982. 10.1172/jci.insight.135982. 32614803 10.1172/jci.insight.135982 PMC7455086

[22] Riemekasten G, Philippe A, Näther M, Slowinski T, Müller DN, Heidecke H, et al. Involvement of functional autoantibodies against vascular receptors in systemic sclerosis. Ann Rheum Dis. 2011;70:530–6. 10.1136/ard.2010.135772. 21081526 10.1136/ard.2010.135772

[23] Li H, Yu X, Cicala MV, Mantero F, Benbrook A, Veitla V, et al. Prevalence of angiotensin II type 1 receptor (AT1R)-activating autoantibodies in primary aldosteronism. J Am Soc Hypertens. 2015;9:15–20. 10.1016/j.jash.2014.10.009. 25537460 10.1016/j.jash.2014.10.009 PMC4314451

[24] Tona F, Civieri G, Vadori M, Masiero G, Iop L, Marra MP, et al. Association of angiotensin II receptor type 1 and endothelin-1 receptor type A agonistic autoantibodies with adverse remodeling and cardiovascular events after acute myocardial infarction. J Am Heart Assoc. 2024;13:e032672. 10.1161/JAHA.123.032672. 38348777 10.1161/JAHA.123.032672 PMC11010093

[25] Williams TA, Jaquin D, Burrello J, Philippe A, Yang Y, Rank P, et al. Diverse responses of autoantibodies to the angiotensin II type 1 receptor in primary aldosteronism. Hypertension. 2019;74:784–92. 10.1161/HYPERTENSIONAHA.119.13156. 31476909 10.1161/HYPERTENSIONAHA.119.13156

[26] Labandeira CM, Pedrosa MA, Quijano A, Valenzuela R, Garrido-Gil P, Sanchez-Andrade M, et al. Angiotensin type-1 receptor and ACE2 autoantibodies in Parkinson´s disease. npj Parkinsons Dis. 2022;8:76. 10.1038/s41531-022-00340-9. 35701430 10.1038/s41531-022-00340-9 PMC9198025

[27] López-Otín C, Blasco MA, Partridge L, Serrano M, Kroemer G. Hallmarks of aging: an expanding universe. Cell. 2023;186:243–78. 10.1016/j.cell.2022.11.001. 36599349 10.1016/j.cell.2022.11.001

[28] Szinger D, Berki T, Németh P, Erdo-Bonyar S, Simon D, Drenjančević I, et al. Following natural autoantibodies: further immunoserological evidence regarding their silent plasticity and engagement in immune activation. Int J Mol Sci. 2023;24:14961. 10.3390/ijms241914961. 37834409 10.3390/ijms241914961 PMC10573785

[29] Cabral-Marques O, Moll G, Catar R, Preuß B, Bankamp L, Pecher AC, et al. Autoantibodies targeting G protein-coupled receptors: an evolving history in autoimmunity. Report of the 4th international symposium. Autoimmun Rev. 2023;22:103310. 10.1016/j.autrev.2023.103310. 36906052 10.1016/j.autrev.2023.103310

[30] Weigold F, Günther J, Pfeiffenberger M, Cabral-Marques O, Siegert E, Dragun D, et al. Antibodies against chemokine receptors CXCR3 and CXCR4 predict progressive deterioration of lung function in patients with systemic sclerosis. Arthritis Res Ther. 2018;20:52. 10.1186/s13075-018-1545-8. 29566745 10.1186/s13075-018-1545-8 PMC5863842

[31] Paniskaki K, Konik MJ, Anft M, Meister TL, Marheinecke C, Pfaender S, et al. Superior humoral immunity in vaccinated SARS-CoV-2 convalescence as compared to SARS-COV-2 infection or vaccination. Front Immunol. 2022;13:1031254. 10.3389/fimmu.2022.1031254. 36389833 10.3389/fimmu.2022.1031254 PMC9659602

[32] Rutledge J, Oh H, Wyss-Coray T. Measuring biological age using omics data. Nat Rev Genet. 2022;23:715–27. 10.1038/s41576-022-00511-7. 35715611 10.1038/s41576-022-00511-7 PMC10048602

[33] Fong S, Pabis K, Latumalea D, Dugersuren N, Unfried M, Tolwinski N, et al. Principal component-based clinical aging clocks identify signatures of healthy aging and targets for clinical intervention. Nat Aging. 2024;4:1137–52. 10.1038/s43587-024-00646-8. 38898237 10.1038/s43587-024-00646-8 PMC11333290

[34] Porter HL, Brown CA, Roopnarinesingh X, Giles CB, Georgescu C, Freeman WM, et al. Many chronological aging clocks can be found throughout the epigenome: implications for quantifying biological aging. Aging Cell. 2021;20:e13492. 10.1111/acel.13492. 34655509 10.1111/acel.13492 PMC8590098

[35] Chen X, Zhong J, Lv Y, Wei L, Zhou H, Yang Y, et al. Epigenetic age acceleration mediates the association between low-grade systemic inflammation and cardiovascular diseases: insight from the NHANES 1999-2002. Clin Epigenetics. 2025;17:89. 10.1186/s13148-025-01895-z. 40450302 10.1186/s13148-025-01895-z PMC12125769

[36] Levine ME, Lu AT, Quach A, Chen BH, Assimes TL, Bandinelli S, et al. An epigenetic biomarker of aging for lifespan and healthspan. Aging (Albany NY). 2018;10:573–91. 10.18632/aging.101414. 29676998 10.18632/aging.101414 PMC5940111

[37] Teschendorff AE, Horvath S. Epigenetic ageing clocks: statistical methods and emerging computational challenges. Nat Rev Genet. 2025;26:350–68. 10.1038/s41576-024-00807-w. 39806006 10.1038/s41576-024-00807-w

[38] Ruan Z, Li D, Huang D, Liang M, Xu Y, Qiu Z, et al. Relationship between an ageing measure and chronic obstructive pulmonary disease, lung function: a cross-sectional study of NHANES, 2007-2010. BMJ Open. 2023;13:e076746. 10.1136/bmjopen-2023-076746. 37918922 10.1136/bmjopen-2023-076746 PMC10626813

[39] Gilmore W, Lund BT, Li P, Levy AM, Kelland EE, Akbari O, et al. Repopulation of T, B, and NK cells following alemtuzumab treatment in relapsing-remitting multiple sclerosis. J Neuroinflammation. 2020;17:189. 10.1186/s12974-020-01847-9. 32539719 10.1186/s12974-020-01847-9 PMC7296935

[40] Groom JR, Luster AD. CXCR3 in T cell function. Exp Cell Res. 2010;317:620–31. 10.1016/j.yexcr.2010.12.017. 10.1016/j.yexcr.2010.12.017 PMC3065205 21376175

[41] Souza FDS, Freitas NL, Gomes YCP, Torres RC, Echevarria-Lima J, da Silva-Filho IL, et al. Following the clues: usefulness of biomarkers of neuroinflammation and neurodegeneration in the investigation of HTLV-1-associated myelopathy progression. Front Immunol. 2021;12:737941. 10.3389/fimmu.2021.737941. 34764955 10.3389/fimmu.2021.737941 PMC8576432

[42] Maurice NJ, McElrath MJ, Andersen-Nissen E, Frahm N, Prlic M. CXCR3 enables recruitment and site-specific bystander activation of memory CD8+ T cells. Nat Commun. 2019;10:4987. 10.1038/s41467-019-12980-2. 31676770 10.1038/s41467-019-12980-2 PMC6825240

[43] Ozga AJ, Chow MT, Lopes ME, Servis RL, Di Pilato M, Dehio P, et al. CXCL10 chemokine regulates heterogeneity of the CD8+ T cell response and viral set point during chronic infection. Immunity. 2022;55:82-97.e8. 10.1016/j.immuni.2021.11.002. 34847356 10.1016/j.immuni.2021.11.002 PMC8755631

[44] Wang X, Zhang Y, Wang S, Ni H, Zhao P, Chen G, et al. The role of CXCR3 and its ligands in cancer. Front Oncol. 2022;12:1022688. 10.3389/fonc.2022.1022688. 36479091 10.3389/fonc.2022.1022688 PMC9720144

[45] Shome M, Chung Y, Chavan R, Park JG, Qiu J, LaBaer J. Serum autoantibodyome reveals that healthy individuals share common autoantibodies. Cell Rep. 2022;39:110873. 10.1016/j.celrep.2022.110873. 35649350 10.1016/j.celrep.2022.110873 PMC9221390

[46] Sitcharungsi R, Ananworanich J, Vilaiyuk S, Apornpong T, Bunupuradah T, Pornvoranunt A, et al. Nephelometry determined serum immunoglobulin isotypes in healthy Thai children aged 2-15 years. Microbiol Immunol. 2012;56(2):117–22. 10.1111/j.1348-0421.2011.00413.x. 22181033 10.1111/j.1348-0421.2011.00413.x

[47] Løk M, Dandanell FE, Frithioff-Bøjsøe C, Lund MAV, Fraulund MM, Lausten-Thomsen U, et al. Reference intervals for serum immunoglobulin A, G, and M in a Danish paediatric population-based cohort. Clin Biochem. 2025;137:110923. 10.1016/j.clinbiochem.2025.110923. 40174761 10.1016/j.clinbiochem.2025.110923

[48] Schauer U, Stemberg F, Rieger CH, Borte M, Schubert S, Riedel F, et al. IgG subclass concentrations in certified reference material 470 and reference values for children and adults determined with the binding site reagents. Clin Chem. 2003;49:1924–9. 10.1373/clinchem.2003.022350. 14578325 10.1373/clinchem.2003.022350

[49] Ma S, Ji Z, Zhang B, Geng L, Cai Y, Nie C, et al. Spatial transcriptomic landscape unveils immunoglobin-associated senescence as a hallmark of aging. Cell. 2024;187:7025-7044.e34. 10.1016/j.cell.2024.10.019. 39500323 10.1016/j.cell.2024.10.019

[50] Pichilingue-Reto P, Raj P, Li QZ, Dozmorov I, Karp DR, Wakeland EK, et al. Serum IgG profiling of toddlers reveals a subgroup with elevated seropositive antibodies to viruses correlating with increased vaccine and autoantigen responses. J Clin Immunol. 2021;41:1031–47. 10.1007/s10875-021-00993-w. 33656624 10.1007/s10875-021-00993-w PMC7927113

[51] Blanco E, Pérez-Andrés M, Arriba-Méndez S, Contreras-Sanfeliciano T, Criado I, Pelak O, et al. Age-associated distribution of normal B-cell and plasma cell subsets in peripheral blood. J Allergy Clin Immunol. 2018;141:2208-2219.e16. 10.1016/j.jaci.2018.02.017. 29505809 10.1016/j.jaci.2018.02.017

[52] Ghraichy M, Galson JD, Kovaltsuk A, von Niederhäusern V, Pachlopnik-Schmid J, Recher M, et al. Maturation of the human immunoglobulin heavy chain repertoire with age. Front Immunol. 2020;11:1734. 10.3389/fimmu.2020.01734. 32849618 10.3389/fimmu.2020.01734 PMC7424015

[53] Yin J, Ibrahim S, Petersen F, Yu X. Autoimmunomic signatures of aging and age-related neurodegenerative diseases are associated with brain function and ribosomal proteins. Front Aging Neurosci. 2021;13:679688. 10.3389/fnagi.2021.679688. 34122052 10.3389/fnagi.2021.679688 PMC8192960

[54] Civieri G, Iop L, Tona F. Antibodies against angiotensin II type 1 and endothelin 1 type A receptors in cardiovascular pathologies. Int J Mol Sci. 2022;23:927. 10.3390/ijms23020927. 35055116 10.3390/ijms23020927 PMC8778295

[55] Philippe A, Kleinau G, Gruner JJ, Wu S, Postpieszala D, Speck D, et al. Molecular effects of auto-antibodies on angiotensin II type 1 receptor signaling and cell proliferation. Int J Mol Sci. 2022;23:3984. 10.3390/ijms23073984. 35409344 10.3390/ijms23073984 PMC8999261

[56] Hegner B, Kretzschmar T, Zhu N, Kleinau G, Zhao H, Kamhieh-Milz J, et al. Autoimmune activation and hypersensitization of the AT1 and ETA receptors contributes to vascular injury in scleroderma renal crisis. Rheumatology (Oxford). 2023;62:2284–93. 10.1093/rheumatology/keac594. 36227102 10.1093/rheumatology/keac594

[57] Speck D, Kleinau G, Szczepek M, Kwiatkowski D, Catar R, Philippe A, et al. Angiotensin and endothelin receptor structures with implications for signaling regulation and pharmacological targeting. Front Endocrinol. 2022;13:880002. 10.3389/fendo.2022.880002. 10.3389/fendo.2022.880002 PMC9063481 35518926

[58] Cabral-Marques O, Halpert G, Schimke LF, Ostrinski Y, Vojdani A, Baiocchi GC, et al. Autoantibodies targeting GPCRs and RAS-related molecules associate with COVID-19 severity. Nat Commun. 2022;13:1220. 10.1038/s41467-022-28905-5. 35264564 10.1038/s41467-022-28905-5 PMC8907309

[59] Lind L, Svensson A, Thörn K, Krzyzowska M, Eriksson K. CD8+ T cells in the central nervous system of mice with herpes simplex infection are highly activated and express high levels of CCR5 and CXCR3. J Neurovirol. 2021;27:145–53. 10.1007/s13365-020-00940-2. 33492607 10.1007/s13365-020-00940-2 PMC7831625

[60] Karin N. CXCR3 ligands in cancer and autoimmunity, chemoattraction of effector T cells, and beyond. Front Immunol. 2020;11:976. 10.3389/fimmu.2020.00976. 32547545 10.3389/fimmu.2020.00976 PMC7274023

[61] Bitsch A, Schuchardt J, Bunkowski S, Kuhlmann T, Brück W. Acute axonal injury in multiple sclerosis. correlation with demyelination and inflammation. Brain. 2000;123:1174–83. 10.1093/brain/123.6.1174. 10825356 10.1093/brain/123.6.1174

