# Supplementary material for: G protein-coupled receptor autoantibody expression patterns in adults with decelerated biological aging mirror pediatric profiles
Source: BMC Immunol. 2026 Jun 4;27:45. doi: 10.1186/s12865-026-00862-4 (PMC13235001; doi:10.1186/s12865-026-00862-4)
Supplement: Supplementary file 1 — Supplementary Material 1. [file 12865_2026_862_MOESM1_ESM.docx]

Supplementary Material

**Table S1:** Autoantibodies dataset and full name

| Autoantibodies dataset | Full name |
| --- | --- |
| G protein-coupled receptors | |
| AT1R | angiotensin II receptor type 1 |
| AT2R | angiotensin II receptor type 2 |
| ADRB2 | beta-2 adrenergic receptor |
| ADRA1A | alpha-1 adrenergic receptor |
| CXCR3 | chemokine (C-X-C motif receptor 3 |
| ETAR or EDNRA | endothelin receptor type A |
| ETBR or EDNRB | endothelin receptor type B |
| MAS1 | mitochondrial assembly protein 1 |
| M5 or CHRM5 | cholinergic receptor muscarinic 5 |
| PAR1 | Protease-activated receptor 1 |
| PAR2 | Protease-activated receptor 2 |
| BDKRB1 | Bradykinin receptor B1 |
| Scavenger receptors (transmembrane receptors) | |
| STAB1 | stabilin-1 receptor |
| ANXA2R | annexin A2 receptor |
| Proteins | |
| ACE2 | angiotensin converting enzyme 2 |
| ANXA2 | annexin A2 |

**Table S2:** Fluorochrome coupled antibodies and fluorescent dye for analysis of CXCR3+ T cells

| Antibodies or fluorescent dye | Fluorochrome | Source | Cat. Nr. |
| --- | --- | --- | --- |
| Fixable Viability-Dye | eFluor780 | eBioscience | 65-0865-14 |
| anti CD3 (clone OKT3) | BV785 | BioLegend | 317330 |
| anti CD4 (clone OKT4) | A700 | BioLegend | 317426 |
| anti CD8 (clone RPA-T8) | V500 | BD Biosciences | 560775 |
| anti CD183 (CXCR3) (clone G025H7) | BV605 | BioLegend | 353728 |


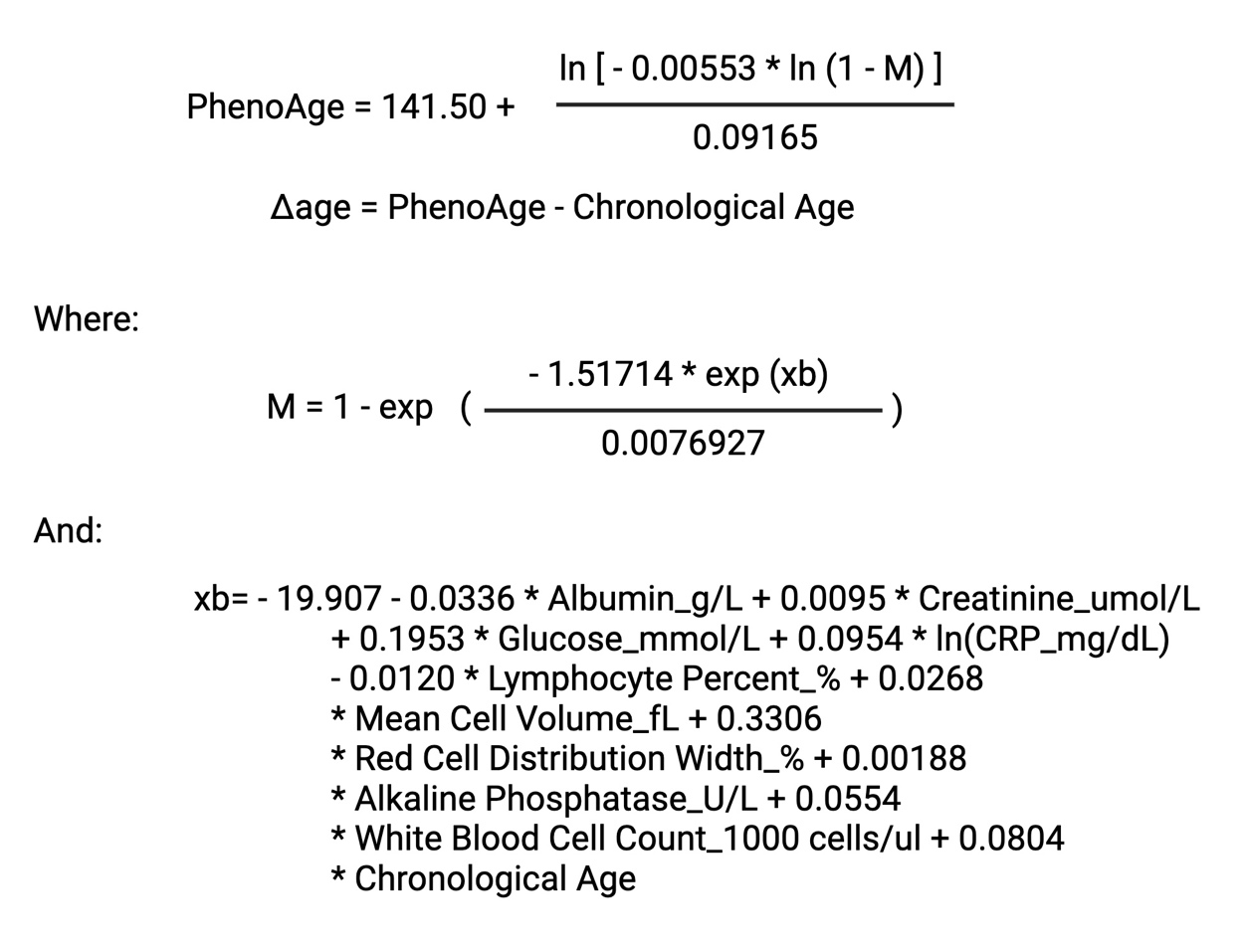


**Figure S1.** **Mathematical equation used to calculate PhenoAge.**

**
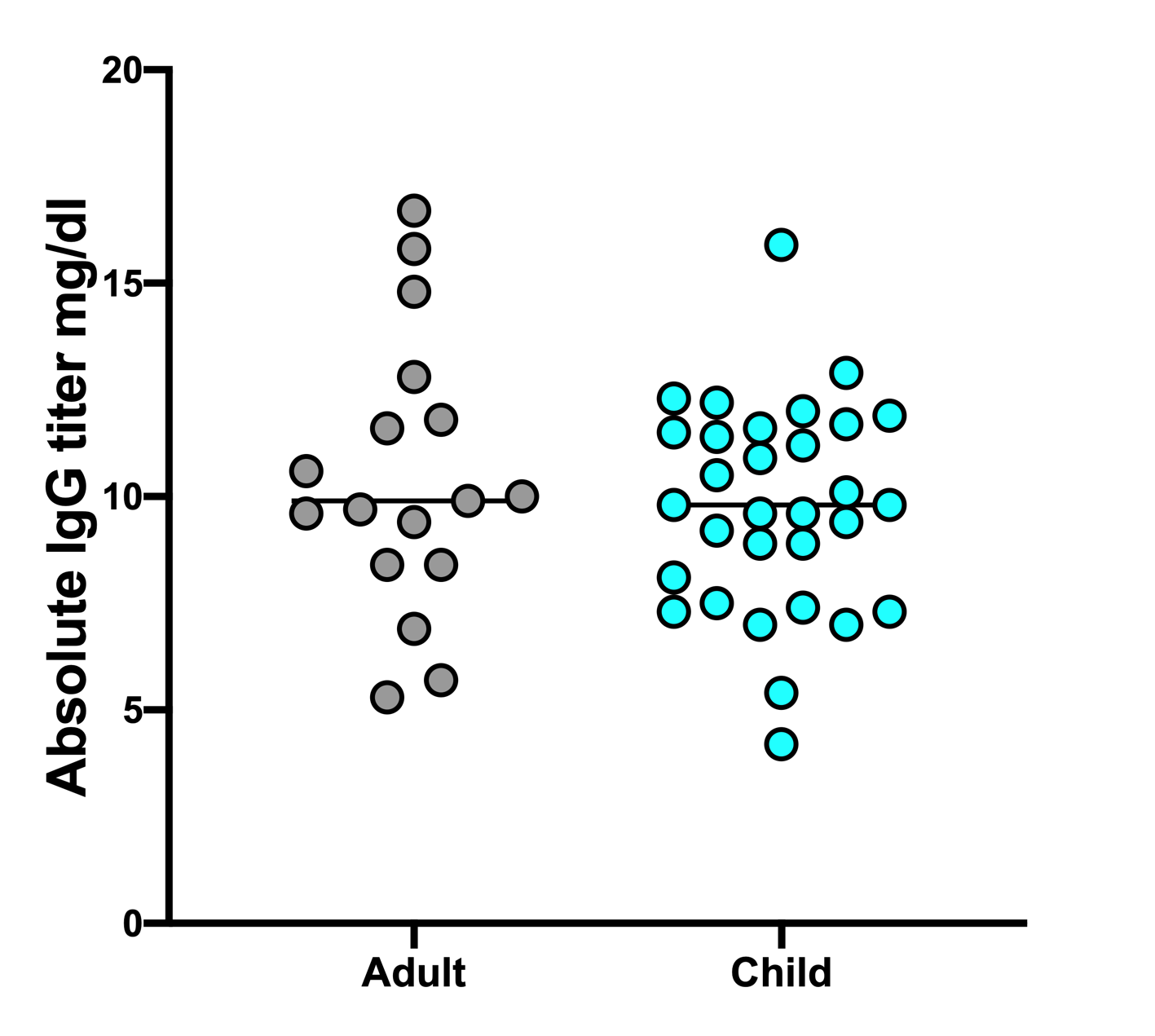
**

**Figure S2.** **Absolute IgG concentrations among adults (n=17) and children(n=31).**


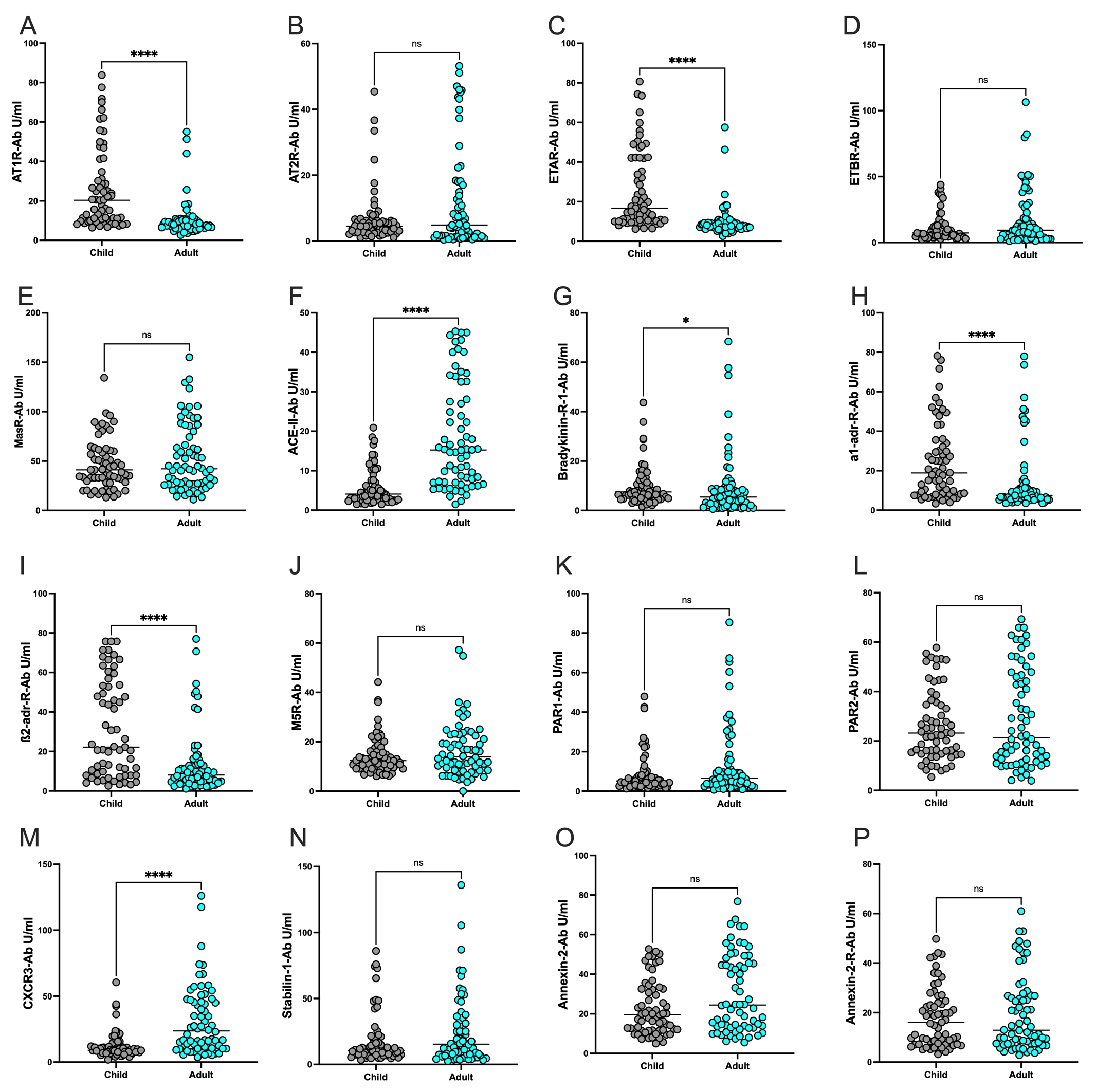


**Figure S3. Comparison of anti-GPCR Aab among adults and children.** Analysis of anti-GPCR Aab concentrations of both study groups. Scatterplots show line at median. Unpaired data were compared with Mann-Whitney-test. p<0.05 was considered significant, only significant p values are documented in the figures.


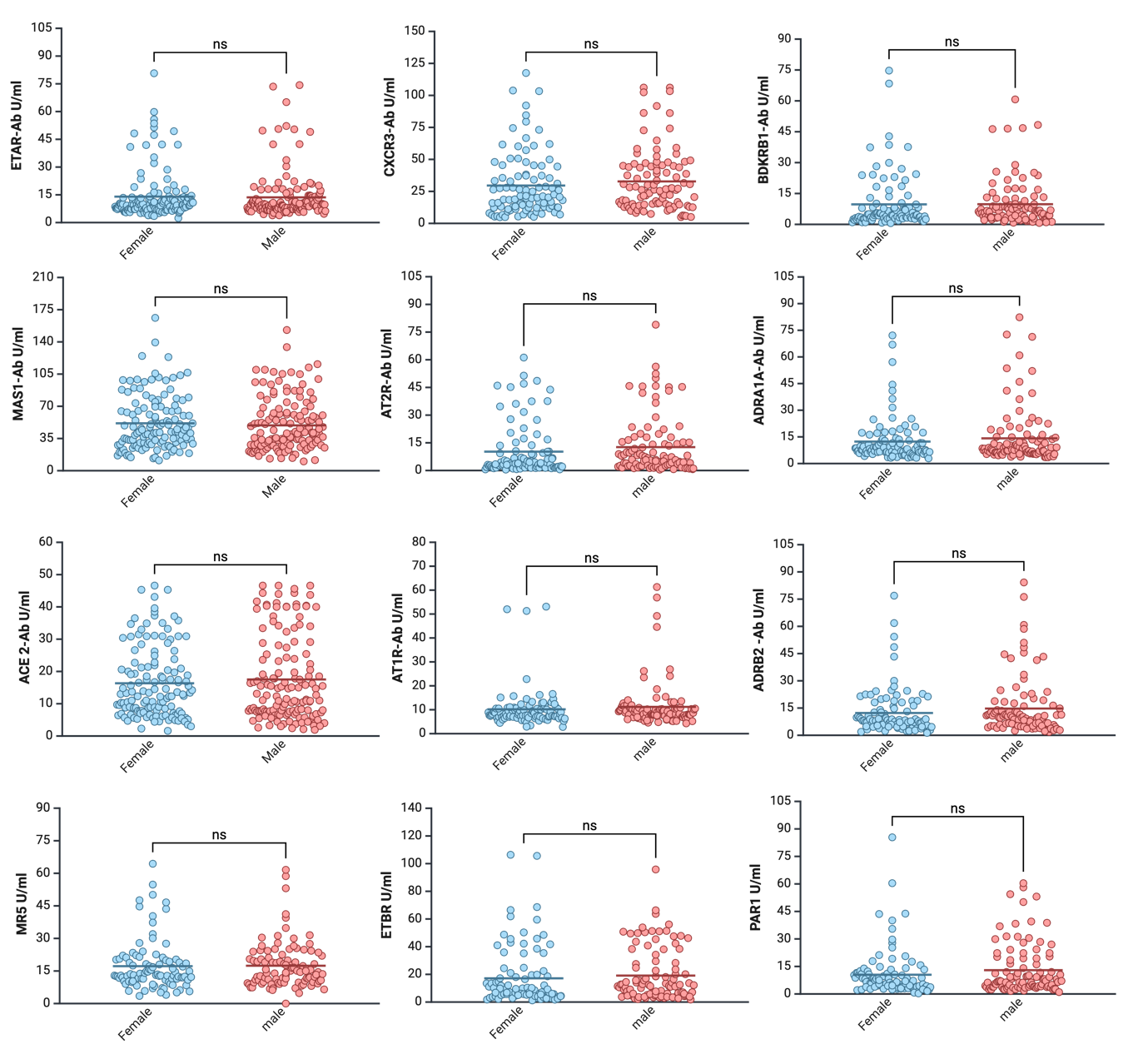


**Figure S4. Comparison of anti-GPCR Aab among female and male participants.** Analysis of anti-GPCR Aab concentrations of both study groups. Scatterplots show line at median. Unpaired data were compared with Mann-Whitney-test. p<0.05 was considered significant, only significant p values are documented in the figures.


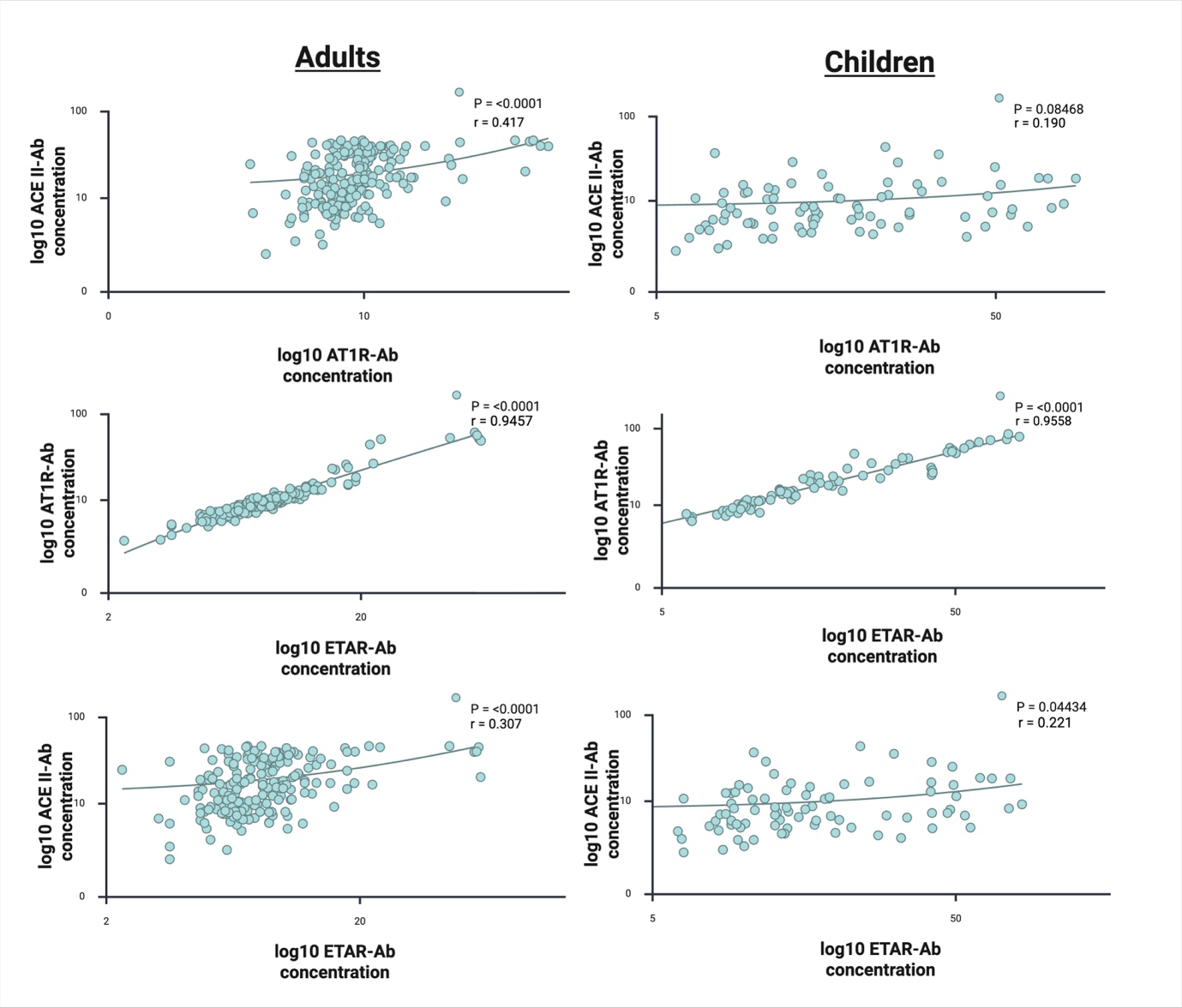


**Figure S5.** Correlation analyses of antibody concentrations (2 antibodies at a time) across four subcohorts: adults, children, adults with +Δage, and adults with −Δage. Figure S5 presents six representative plots illustrating these correlations.


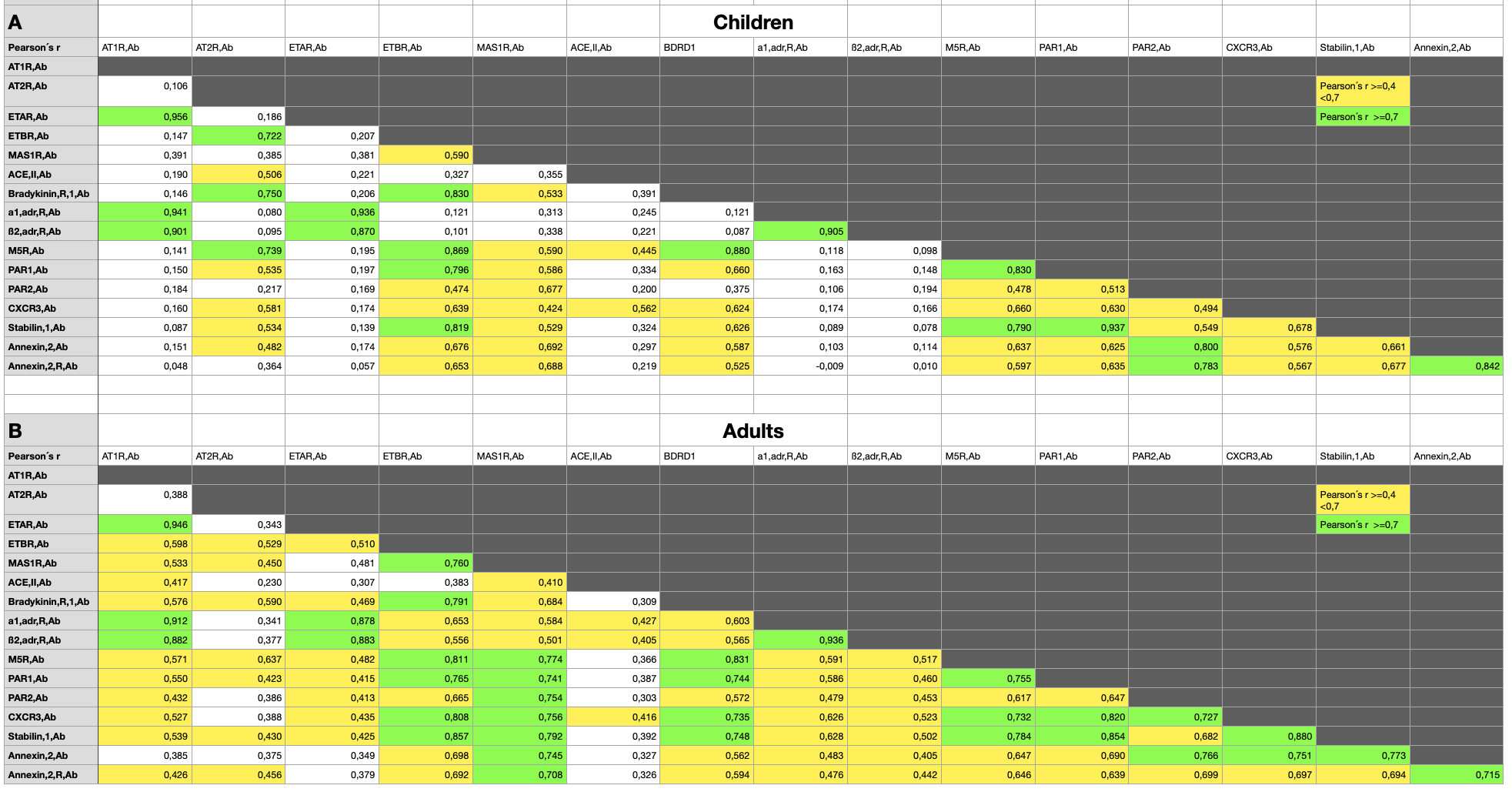


**Figure S6. Correlation of diverse anti-GPCR Aab based on chronological age.**

A. Correlations of diverse anti-GPCR Aab among children. B. Correlations of diverse anti-GPCR Aab among children. Moderate correlations are shown in yellow, while strong correlations are shown in green.


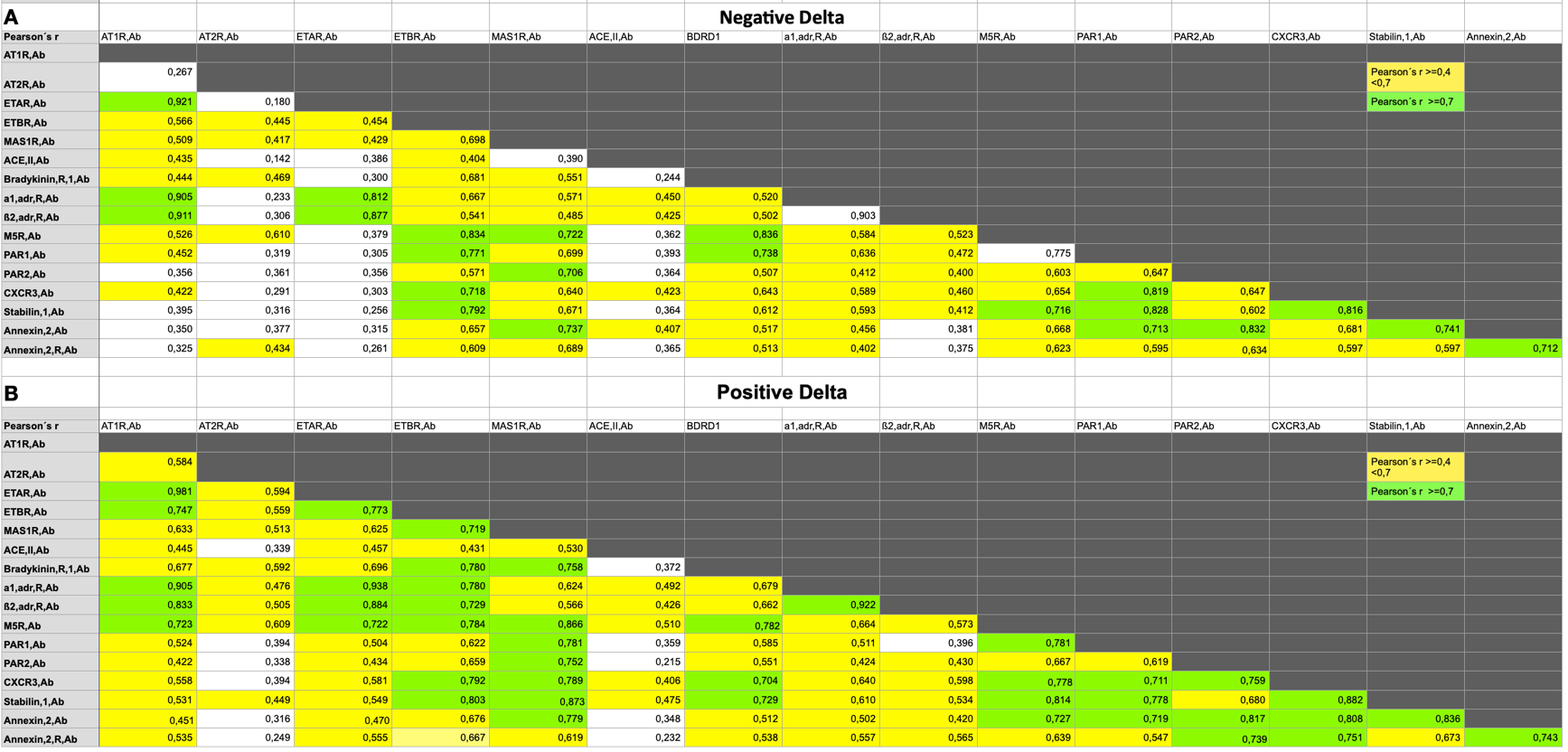


**Figure S7.** **Correlation of diverse anti-GPCR Aab based on age gap (Δ age).**

A. Correlations of diverse anti-GPCR Aab among adults with negative Δ age. B. Correlations of diverse anti-GPCR Aab among adults with positive Δ age. Moderate correlations are shown in yellow, while strong correlations are shown in green.
